# Social and emotional difficulties in children with ADHD and the impact on school attendance and healthcare utilization

**DOI:** 10.1186/1753-2000-6-33

**Published:** 2012-10-04

**Authors:** Peter Classi, Denái Milton, Sarah Ward, Khaled Sarsour, Joseph Johnston

**Affiliations:** 1 Eli Lilly and Company, Indianapolis, IN, USA; 2 Global Health Outcomes – Neuroscience, Eli Lilly and Company, Indianapolis, IN, USA

**Keywords:** Comorbidities, Attention deficit hyperactivity disorder, Resource use, Outcomes

## Abstract

**Background:**

The objective of this study was to examine the impact of co-occurring social and emotional difficulties on missed school days and healthcare utilization among children with attention deficit/hyperactivity disorder (ADHD).

**Methods:**

Data were from the 2007 U.S. National Health Interview Survey (NHIS) and were based on parental proxy responses to questions in the Sample Child Core, which includes questions on demographics, health, healthcare treatment, and social and emotional status as measured by questions about depression, anxiety, and phobias, as well as items from the brief version of the Strength and Difficulties Questionnaire (SDQ). Logistic regression was used to assess the association between co-occurring social and emotional difficulties with missed school days and healthcare utilization, adjusting for demographics.

**Results:**

Of the 5896 children aged 6–17 years in the 2007 NHIS, 432 (7.3%) had ADHD, based on parental report. Children with ADHD and comorbid depression, anxiety, or phobias had significantly greater odds of experiencing > 2 weeks of missed school days, ≥ 6 visits to a healthcare provider (HCP), and ≥ 2 visits to the ER, compared with ADHD children without those comorbidities (OR range: 2.1 to 10.4). Significantly greater odds of missed school days, HCP visits, and ER visits were also experienced by children with ADHD who were worried, unhappy/depressed, or having emotional difficulties as assessed by the SDQ, compared with ADHD children without those difficulties (OR range: 2.2 to 4.4).

**Conclusions:**

In children with ADHD, the presence of social and emotional problems resulted in greater odds of missed school days and healthcare utilization. These findings should be viewed in light of the limited nature of the parent-report measures used to assess social and emotional problems.

## Background

Attention-deficit/hyperactivity disorder (ADHD) is a common neuropsychiatric condition in children [1-5] with an estimated prevalence of 3 to 7% [1]. Attention-deficit/hyperactivity disorder is characterized by symptoms of inattention and/or hyperactivity-impulsivity that are more frequently displayed and more severe than typically observed in individuals at a comparable level of development [1], are usually evident in more than one setting (e.g., home and school), and result in impairment in multiple domains of functioning [3,6,7]. A rich literature speaks to the burden that ADHD imposes on patients, families, and society as a whole, including negative effects on individual educational [8,9] and social outcomes [3,6], negative effects on patient and parent quality of life [7], and increased utilization of and spending on healthcare services [10-17].

Social and emotional difficulties are particularly common and problematic in children with ADHD. Social difficulties present in a variety of forms and can lead to conflicts with family and problems with peers [18-21]. Emotional difficulties often include poor emotional self-regulation, aggression, and reduced empathy [22,23]. It should be noted that these challenges exist on a continuum. Relatively mild difficulties may fail to come to clinical attention, while in other cases such difficulties can contribute to overt, physician-diagnosed, comorbid mental health disorders, including anxiety, depression, and conduct disorder [22-24]. Major depressive disorder has been reported to occur in 12-50% of children with ADHD in community samples [25-27], and anxiety disorder, established by formal diagnostic interview, was comorbid in one third of ADHD patients enrolled in the commonly cited Multimodal Treatment Study of Children With ADHD [28]. Comorbid mental health conditions, including anxiety and depression, are extremely typical among children with ADHD and have been shown to be associated with greater functional impairment and worse educational outcomes [29-33].

Given the above data, it is important to understand the ways in which co-occurring conditions, including those characterized as social and emotional difficulties, can lead to various types of poor outcomes and functional impairment in children with ADHD, so that caregivers and providers can target interventions appropriately. In this study, we used data from the United States (U.S.) National Health Interview Survey (NHIS) to explore the association between social and emotional difficulties in children with ADHD and select outcomes. Available measures included both parent report of social and emotional difficulties (the brief version of the Strength and Difficulties Questionnaire [SDQ]) and parent report of physician-diagnosed depression, anxiety, and phobias. Unfortunately, teacher ratings and physician diagnoses were not available, and thus independent validation of parent reports was not possible. Available outcomes of interest included school days missed and emergency room (ER) and healthcare provider (HCP) visits over the past 12 months. We hypothesized that the presence of social and emotional difficulties in children with ADHD would be associated with increased school absenteeism and increased healthcare utilization, compared to ADHD children without these difficulties.

## Methods

### Data

The data were from a subset of the publicly available 2007 NHIS [4,34]. The NHIS is an annual cross-sectional survey designed to capture health-related trends in a sample representative of the civilian, non-institutionalized population of the U.S.; these data can be weighted to represent the U.S. population [35,36]. The sampling plan for the NHIS followed a multistage area probability design and oversampled African Americans, Hispanics, and Asians. Data were collected by trained interviewers from the U.S. Census Bureau who visited each selected household and administered the NHIS in person. Interviewers collected basic health and socio-demographic information on all household members, and gathered more extensive information on one sample adult and one sample child per family. An adult from the household, typically the child’s parent, served as the proxy respondent for each child. Of the 10,658 children under 18 years of age eligible for the Sample Child Core questionnaire, the NHIS 2007 survey obtained data from 9417 sample children with a conditional response rate of 88.4%.

### Measures

Data analyzed in the current study are from the Sample Child Core of the 2007 NHIS [37], which includes questions on demographics, health, healthcare treatment, healthcare access, healthcare utilization, and social and emotional status. All information was obtained based on parental/adult proxy reports. Demographic information was collected on gender, age, race, family income, and health insurance status.

ADHD status was ascertained based on the parent reporting whether they had ever been told by a doctor or healthcare professional that their “child had Attention Deficit Hyperactivity Disorder (ADHD) or Attention Deficit Disorder (ADD).” The presence of depression, phobias, and anxiety, respectively, were defined based on the parent’s responses to the following 3 questions: “During the past 12 months, has a doctor or other health professional told you that your child had: (1) depression, (2) phobias or fears, (3) anxiety or stress?” For each child, an incremental internalizing burden index was computed by adding the number of internalizing problems (i.e., depression, anxiety, phobias) they experienced.

Parental reports of their child’s social and emotional difficulties were also defined using items from the brief version SDQ [38,39]. The SDQ is a 25-item behavioral screening questionnaire for 4–17 year olds and includes five scales, each with five-items that assess the following domains: Emotional symptoms, conduct problems, hyperactivity/inattention, peer relationship problems, and prosocial behavior. The SDQ has demonstrated evidence of validity and reliability [40]. The 2007 NHIS included 6 questions from the SDQ, which asked parents to report whether, over the preceding 6-month period, their child: 1) was well behaved, usually did what adults requested; 2) had many worries, or often seemed worried; 3) was often unhappy, depressed or tearful; 4) got along better with adults than with other children; 5) had good attention span, sees chores or homework through to the end; and 6) had difficulties in any of the following areas: emotions, concentration, behavior, or being able to get along with other people. Responses were dichotomized based on positive (“somewhat true” and “certainly true”) versus negative (“not true”) responses.

Two questions were used to define HCP and ER visits in the preceding 12 months. Parents reported on the number of times the child had “seen a doctor or other healthcare professional about his/her health at a doctor’s office, a clinic, or some other place.” The responses were dichotomized into < 6 versus ≥ 6 visits (i.e., on average, ≥ 1 HCP visit every other month). Parents also reported on the number of times the child had “visited a hospital emergency room (ER) about his/her health.” The responses were dichotomized into < 2 versus ≥ 2 visits to the ER (i.e., on average, ≥ 1 ER visit every 6 months). School attendance was based on parental reports on the number of days their child “missed from school because of illness or injury in the past 12 months.” The responses were dichotomized as having missed < 2 or ≥ 2 weeks (i.e., 10 days) of school.

### Sample construction

The analyses for the current study included children aged 6–17 years whose adult proxy answered the ADHD diagnosis question in the NHIS 2007 survey. Children and adolescents less than 6 years of age (n=3284); those with mental retardation, developmental delay, or autism (n=230); and those who were missing the ADHD status variable (n=7) were excluded. The final sample included 5896 children and adolescents, including 432 with ADHD. The 5464 children and adolescents without ADHD were included in some of the secondary analyses.

### Analyses

The primary analysis for this study was focused on the association between co-occurring social and emotional difficulties with missed school days and healthcare utilization among children with ADHD. To assess this association, logistic regression models with dichotomized outcomes (i.e., missed school days, HCP visits, ER visits) as dependent measures and comorbid condition (e.g., depression, incremental internalizing burden index, SDQ items) as independent measures adjusting for gender, age category (6–11 years [children], 12–17 years [adolescents]), race, income, insurance status, and ADHD medication-use were employed.

To give context to the primary analysis and determine if there was a differential association between comorbid conditions on missed school days and healthcare utilization by ADHD status, logistic regression models with outcome as the dependent measure and ADHD status, comorbid condition, and ADHD status-by-comorbid condition interaction as independent measures adjusting for gender, age category, race, income, and insurance status were utilized. Finally, descriptive statistics were used to characterize the ADHD and non-ADHD subsamples.

All analyses were conducted in SAS version 9.1 (SAS Institute Inc., NC), using procedures specifically designed to properly analyze complex survey data which employ sample weight, stratification, and cluster information. All percentages, means, and estimates were adjusted to account for the NHIS survey design. All statistical tests of differences in independent measures, including interactions, were conducted using a 2-sided significance level of 0.05.

## Results

Of the 5896 children aged 6–17 years in the 2007 NHIS, 432 (7.3%) had ADHD based on parental reports. The majority of children with ADHD were male (69.7%), adolescent (65.9%), white (75.5%), insured (91.3%), and with a family income less than $75,000 per year (68.8%). Sixty-eight percent of these children with ADHD had been and/or were currently being treated with a prescription medication to treat difficulties with concentration, hyperactivity, or impulsivity. Approximately one-third of these ADHD children had comorbid anxiety, while comorbid depression (16.5%) and phobias (7.2%) were less common. Compared with a reported formal diagnosis, a higher percentage of parents reported that their ADHD child was unhappy or depressed (27.4%) or often seemed worried (47.3%). Similarly, about 40% reported their ADHD child got along better with adults than with children and did not have good attention, while about one-third reported their child had difficulties in emotions, concentration, behavior, or being able to get along with other people. Despite this data, over 90% of parents of ADHD children reported that their child was generally well behaved (Table 1). Descriptive statistics are also provided in Table 1 for the non-ADHD sample.

**Table 1:** Descriptive statistics

**Characteristics**	**ADHD (n=432)**	**non-ADHD (n=5464)**
**Gender, n (%)**		
Male	309 (69.7)	2717 (48.8)
**Age, n (%)**		
6-11 years	153 (34.1)	2531 (49.5)
12-17 years	279 (65.9)	2933 (50.5)
**Race, n (%)**		
White	317 (75.5)	3956 (76.1)
Black	79 (16.2)	966 (15.5)
Asian	6 (1.0)	292 (4.0)
Other	30 (7.3)	242 (4.4)
**Family Income, n (%)**		
$0 - $34,999	140 (33.1)	1637 (29.5)
$35,000 - $74,999	132 (35.7)	1641 (33.4)
$75,000 - $99,999	52 (13.5)	647 (14.5)
$100,000 and over	73 (17.7)	1006 (22.6)
**Medical Insurance, n (% yes)**	391 (91.3)	4801 (89.8)
**Medication Ever Prescribed for Difficulties with Concentration, Hyperactivity, or Impulsivity, n (% yes)**	278 (67.6)	39 (0.8)
**Outcome Measures, n (% yes)**		
≥ 2 ER visits past 12 months	40 (11.1)	286 (5.6)
≥ 6 Doctor or HCP visits past 12 months	122 (30.9)	481 (9.4)
≥ 2 weeks of school missed in past 12 months	41 (8.4)	204 (3.6)
**Social and Emotional Difficulties, n (% yes)**		
Anxiety/stress in past 12 months	117 (32.2)	330 (6.3)
Depression in past 12 months	58 (16.5)	106 (2.0)
Phobias/fears in past 12 months	35 (7.2)	111 (1.9)
Strength and Difficulties Questionnaire*, n (%)		
SDQ 1 - Not well behaved	35 (7.0)	133 (2.3)
SDQ 2 - Often seems worried	194 (47.3)	1115 (21.3)
SDQ 3 - Unhappy/depressed	106 (27.4)	533 (9.8)
SDQ 4 - Gets along better with adults than children	196 (42.7)	1706 (31.0)
SDQ 5 - Doesn’t have good attention	186 (44.7)	414 (7.9)
SDQ 6 - Difficulties w/emot/conc/ beh/getting along	126 (32.8)	101 (2.0)

Note: Percents reported are based on weighted frequencies and thus may vary slightly from the expected values based on the reported n’s.

* SDQ1: He/she is generally well behaved, usually does what adults request; SDQ2: He/she has many worries, or often seems worried; SDQ3: He/she is often unhappy, depressed, or tearful; SDQ4: He/she gets along better with adults that with other children/youth; SDQ5: He/she has good attention span, sees chores or homework through to the end; SDQ6: Overall, do you think that [name] has difficulties in any of the following areas: emotions, concentration, behavior, or being able to get along with other people?

Table 2 presents the percentages of missed school days, ER visits, and HCP visits overall and by reported presence of social and emotional difficulties for children with ADHD. In addition, Table 2 presents odds ratios (OR [95% confidence interval (CI)]) that represent the association between co-occurring social and emotional difficulties and missed school days, ER visits, and HCP visits.

**Table 2:** Social and emotional difficulties and SDQ items for subjects with ADHD

	**Missed School Days (>2 weeks)**			**ER Visits (≥2 visits)**			**HCP Visits (≥6 visits)**		
**All ADHD Subjects**	**8.4%**			**11.1%**			**30.9%**		
**Social and Emotional Difficulties**	**Yes (%)**	**No (%)**	**OR [95% CI]**	**Yes (%)**	**No (%)**	**OR [95% CI]**	**Yes (%)**	**No (%)**	**OR [95% CI]**
Anxiety/stress in past 12 months	14.2	5.7	3.4 [2.2, 5.1]	17.4	8.2	2.1 [1.2, 3.6]	51.9	21.4	2.9 [2.0, 4.4]
Depression in past 12 months	25.7	4.8	10.1 [5.7, 17.8]	26.5	7.8	3.5 [2.0, 6.4]	72.3	22.5	7.4 [4.3, 12.7]
Phobias/fears in past 12 months	33.0	6.5	10.4 [4.2, 26.2]	22.6	10.2	2.4 [1.0, 5.4]	53.2	29.2	3.0 [1.3, 7.2]
Strength and Difficulties Questionnaire*	**Yes (%)**	**No (%)**	**OR [95% CI]**	**Yes (%)**	**No (%)**	**OR [95% CI]**	**Yes (%)**	**No (%)**	**OR [95% CI]**
SDQ 1 - Not well behaved	22.4	7.5	5.5 [2.2, 13.8]	30.3	9.5	5.2 [2.0, 13.5]	37.4	30.6	1.4 [0.7, 2.9]
SDQ 2 - Often seems worried	13.4	4.1	3.2 [2.1, 4.8]	16.7	5.7	2.6 [1.4, 4.7]	42.0	21.3	2.2 [1.5, 3.1]
SDQ 3 - Unhappy/depressed	17.0	5.3	3.9 [2.3, 6.4]	19.9	7.6	2.2 [1.3, 3.8]	52.5	23.3	2.6 [1.7, 3.8]
SDQ 4 - Gets along better with adults than children	7.6	9.2	0.7 [0.4, 1.3]	9.9	11.7	0.9 [0.5, 1.6]	29.4	32.1	1.0 [0.6, 1.5]
SDQ 5 - Doesn’t have good attention	12.1	5.7	2.9 [1.8, 4.6]	15.4	7.4	2.5 [1.6, 4.1]	37.9	25.5	1.5 [1.0, 2.4]
SDQ 6 - Difficulties w/emot/conc/beh/ getting along	16.2	4.8	4.4 [2.8, 6.9]	20.2	6.5	3.0 [1.8, 5.0]	54.6	19.4	3.8 [2.6, 5.4]

Note: Interaction effects were significant for ADHD status and SDQ item 4 for missed school days (*P*=0.0490), ADHD status and SDQ item 1 for ER visits (*P*=0.0060), and ADHD status and SDQ item 2 for ER visits (*P*=0.0420).

* SDQ 1: He/she is generally well behaved, usually does what adults request; SDQ 2: He/she has many worries, or often seems worried; SDQ 3: He/she is often unhappy, depressed, or tearful; SDQ 4: He/she gets along better with adults that with other children/youth; SDQ 5: He/she has good attention span, sees chores or homework through to the end; SDQ 6: Overall, do you think that [name] has difficulties in any of the following areas: emotions, concentration, behavior, or being able to get along with other people?

Overall, more ADHD children experienced at least 6 HCP visits (31%), compared with experiencing at least 2 ER visits (11%) and missing more than 2 weeks of school (8%). When assessing the impact of co-occurring social and emotional difficulties on school attendance and healthcare utilization, ADHD children with anxiety had significantly greater odds of missing more than 2 weeks of school (3.4 [2.2, 5.1]), having at least 2 ER visits (2.1 [1.2, 3.6]), and having at least 6 HCP visits (2.9 [2.0, 4.4]), compared with those without anxiety. For ADHD children with depression, those with the comorbid condition were 10 times as likely as those without the comorbid condition to miss more than 2 weeks of school (10.1 [5.7, 17.8]); 7 times as likely to have at least 6 HCP visits (7.4 [4.3, 12.7]); and 3.5 times as likely to have at least 2 ER visits (3.5 [2.0, 6.4)]. Similarly, ADHD children with comorbid phobias were 10 times as likely to miss more than 2 weeks of school (10.4 [4.2, 26.2]) as those without phobias, while being 3 times as likely to have at least 6 HCP visits (3.0 [1.3, 7.2]) and 2 times as likely to have at least 2 ER visits (2.4 [1.0, 5.4]). In addition, with each incremental increase in internalizing burden, ADHD children had significantly greater odds of missing at least 2 weeks of school (3.1 [2.4, 4.0]), having at least 6 HCP visits (2.2 [1.7, 2.8]), and having at least 2 ER visits (1.7 [1.3, 2.2]).

For the single, general SDQ item assessing difficulties in emotions, concentration, behavior, or being able to get along with other people (item 6), ADHD children with at least one of these complications experienced significantly greater odds of missing more than 2 weeks of school (4.4 [2.8, 6.9]), experiencing at least 2 ER visits (3.0 [1.8, 5.0]), and having at least 6 HCP visits (3.8 [2.6, 5.4]), compared with those who did not have these difficulties.

For the SDQ items associated with emotional difficulties (items 2 and 3), ADHD children who were worried had significantly higher odds of missing more than 2 weeks of school (3.2 [2.1, 4.8]), experiencing at least 2 ER visits (2.6 (1.4, 4.7]), and having at least 6 HCP visits (2.2 [1.5, 3.1]), compared with those who were not worried. Likewise, ADHD children who were unhappy/depressed experienced significantly greater odds of missing more than 2 weeks of school (3.9 [2.3, 6.4]), experiencing at least 2 ER visits (2.2 (1.3, 3.8]), and having at least 6 HCP visits (2.6 [1.7, 3.8]), compared with those who were not unhappy/depressed.

For the SDQ items associated with social or behavioral symptoms of ADHD (items 1, 4, and 5), children who did not have good attention were about 3 times as likely as children who did have good attention to miss more than 2 weeks of school (2.9 [1.8, 4.6]) and 2.5 times as likely to have at least 2 ER visits (2.5 [1.6, 4.1]), while experiencing at least 6 HCP visits was similar for those with and without good attention (1.5 [1.0, 2.4]). As observed with good attention, ADHD children who were not well behaved had significantly higher odds of missing more than 2 weeks of school (5.5 [2.2, 13.8]) and experiencing at least 2 ER visits (5.2 (2.0, 13.5]), compared with those who were well behaved, while the odds for having at least 6 HCP visits were similar between those who were well behaved and who were not well behaved (1.4 [0.7, 2.9]). ADHD children who got along better with adults experienced similar odds of missing more than 2 weeks of school (0.7 [0.4, 1.3]), having at least 2 ER visits (0.9 (0.5, 1.6]), and having at least 6 HCP visits (1.0 [0.6, 1.5]), compared with those who did not get along better with adults.

When assessing if there was a differential effect of comorbid condition on missed school days and healthcare utilization by ADHD status, three interactions were significant. As stated above, children with ADHD who got along better with adults had lower odds, although not significant, of missing more than 2 weeks of school compared with ADHD children who did not get along better with adults (0.7 [0.4, 1.3]). Conversely, non-ADHD children who got along better with adults experienced significantly higher odds of missing more than 2 weeks of school (1.8 [1.2, 2.7]), compared with those who did not get along better with adults. This diametric relationship resulted in a significant interaction effect (*P*=0.0490). Significant interactions were also observed for ADHD status and being well behaved (*P*=0.0060), as well as being worried (*P*=0.0420), for children experiencing at least 2 ER visits. ADHD children who were not well behaved had significantly greater odds of having at least 2 ER visits, compared with those who were well behaved (5.2 [2.0, 3.5]), while non-ADHD children who were not well behaved had lower odds of having at least 2 visits to the ER, compared with non-ADHD children who were well behaved (0.5 [0.2, 1.2]). On the other hand, both ADHD and non-ADHD children who worried experienced increased odds of having at least 2 ER visits; however, the comparison was significant for the ADHD cohort (2.6 (1.4, 4.7]) and was not significant for the non-ADHD group (1.2 [0.9, 1.7]).

## Discussion

This study adds to the literature which demonstrates that social and emotional difficulties in children with ADHD can contribute to higher rates of unfavorable outcomes. In particular, these data suggest that both parent-observed child social difficulties (e.g., not being “well behaved”) and emotional difficulties (e.g., worry) and parent report of physician diagnosed affective disorders (e.g., depression) can be used to identify children with significantly elevated rates of school absenteeism and ER and HCP utilization. Strikingly, a positive response on a single general item from the SDQ (i.e., item 6, “had difficulties in any of the following areas: emotions, concentration, behavior, or being able to get along with other people”) identifies a subset of children 3 to 4 times as likely as peers answering negatively, to exhibit all three of the examined adverse outcomes. While this general association is compelling, consideration of the other independent measures provides additional insights. The remaining eight items examined can be organized according to the clinical/psychological domain to which they speak: Three to anxious symptoms (i.e., the SDQ “worry” item and the physician-diagnosed “anxiety or stress” and “phobias or fears” items); two to mood (i.e., the SDQ “unhappy/depressed” item and physician-diagnosed “depression”); and three to core ADHD symptoms or social behavior (i.e., the “well behaved,” “good attention span,” and “got along better with adults” SDQ items).

In general, the presence of anxious symptoms had a more pronounced impact on school absenteeism than on ER or HCP utilization. While no further detail as to the nature of the anxiety was available, anxiety-related school avoidance is a well described phenomenon, and, in this regard, it is notable that of the three items, the strongest relationship with school absenteeism was observed for physician-diagnosed phobias (OR 10.4). It is also interesting that parent observation of “worry” (SDQ item 2) was as strongly predictive of increased absenteeism as was report of physician-diagnosed anxiety (OR 3.2 vs. 3.4, respectively).

Consistent with what has been observed in other studies, parent report of physician-diagnosed depression was associated with worse outcomes [7,41], and it predicted the largest increase in odds of more HCP visits, across all items examined. This result may be due, in part, to the fact that depression is more likely to lead to closer physician follow-up, greater use of pharmacotherapy, and higher rates of specialist referral relative to children with anxiety disorders or phobias, which are generally managed through behavioral therapies. In contrast to the pattern seen with the anxiety items, physician-diagnosed depression was associated with substantially greater odds of both increased school absenteeism (OR 10.1 vs. 3.9) and HCP visits (OR 7.4 vs. 2.6) than was the parental report of a child being “often unhappy, depressed or tearful” (SDQ item 3). These findings suggest that the 16.5% of ADHD children with physician-diagnosed depression are likely a more severely affected subgroup of the 27.4% of children rated positive on SDQ item 3.

The impact of the remaining SDQ items on the outcomes of interest was mixed. A negative response on SDQ item 1 (i.e., “was well behaved, usually did what adults requested”) was actually the strongest predictor of multiple ER visits across all items, and the strongest among SDQ items of school absenteeism; in contrast, poor attention span (SDQ item 5) was more weakly associated with these outcomes. This result is consistent with the fact that children with predominantly inattentive forms of ADHD are more likely to exhibit more subtle problems (e.g., school failure) than their more declarative peers with hyperactivity. Finally, a child’s getting along “better with adults than with other children” (SDQ item 4) did not appear to be associated with any of the outcomes examined, perhaps because of the ambiguous nature of the question (i.e., could be interpreted as a positive or negative attribute). This outcome is further reinforced by the significant interaction between ADHD status and this item in the models that predict school absenteeism (*P*=0.049). For non-ADHD children, those who got along better with adults tended to miss more school than those who did not; while amongst children with ADHD, those who got along better with adults tended to miss less school. One possible explanation for this finding is that, among children with ADHD, peer rejection is the norm [42]; thus, the ability to “get along better with adults than peers” may indicate positive relationships with teachers in a formalized setting. In contrast, this trait may reflect interpersonal or social deficits in children without ADHD that are associated with increased problems at school.

The use of nationally representative survey data from NHIS represents a particular study strength, permitting generalization of findings to the entire U.S. population of children with ADHD. The sampling design enhances validity by ensuring that participants are selected for inclusion in the study independent of their status for the predictor and outcome variables of interest. Another study strength is the use of the brief SDQ, which has been shown to be a reliable and valid screening instrument for child psychiatric disorders [43,44].

Our findings should be interpreted in light of several important considerations regarding the measures available within the NHIS survey. First, relatively few items were available to assess emotional and social difficulties, and indeed no information was available regarding the duration or severity of these problems. Furthermore, measures of both emotional and social difficulties and of the outcomes of interest were based on parent report. The use of direct parent report measures of both independent and dependent measures is both a strength and weakness. On the positive side, it permits the collection of data elements that are not available in secondary sources, such as administrative claims. On the negative side, parent report data are subject to recall bias and are necessarily inferior to school attendance and healthcare claims records for the outcomes of interest. In other studies using the SDQ, investigators have reported greater validity and reliability of estimates of emotional and behavioral problems based on reports from multiple informants including parents, teachers, and, for some age groups, children [44,45]. As parallel assessments from these sources were not available, we were unable to independently verify parents’ assessments. Finally, it should be noted that the thresholds chosen when dichotomizing outcome measures were somewhat arbitrary; these choices are in no way intended to imply that school absences or healthcare utilization above these thresholds were unnecessary or inappropriate in any way.

Several additional study limitations deserve mention. The definition of ADHD status was based on the parent response to a single item. While the validity of this approach is suspect, it should be noted that the prevalence of ADHD in this sample is very close to what would be expected based on estimates from other studies [2,3] and the prevalence reported in the Diagnostic and Statistical Manual of Mental Disorders, Fourth Edition (DSM-IV) [1]. Information about the presence of ADHD and mood disorders in parents was also unavailable. Thus, while these factors could clearly serve as an important source of bias, we were unable to examine their impact on the outcomes assessed. Also, a substantial proportion of the sample (9.6%) was missing data on the income variable and, therefore, those subjects were not included in the analysis. A sensitivity analysis was performed using multiple imputations, and the results were consistent with those presented for the non-imputed samples. It is important to note that the sample size was considerably smaller for ADHD children with a reported diagnosis of depression and phobias, as well as for ADHD children who were not well behaved compared to ADHD children without these social and emotional difficulties. Given this information, the comparisons for these particular difficulties should be interpreted with caution. The NHIS also has very limited information regarding the medications the children were taking at the time of the study and, thus, the analyses did not control for medication status, types of medications, or medication adherence. Finally, the current study reports on the results of a large number of statistical tests in which the *P*-values were not adjusted for multiple comparisons to control the type I error rate. This study was intended to generate hypotheses rather than confirm specific hypotheses. Based on all of the aforementioned limitations, these results would need to be replicated in future studies.

## Conclusions

Our findings provide further evidence that the presence of social and emotional difficulties in children with ADHD contributes to the functional impairment observed in this population. In particular, children manifesting these problems are more likely to experience greater school absenteeism and to incur more ER and HCP visits than their unaffected peers. Greater awareness of these associations, together with focused efforts to identify and manage these children appropriately, could lead to improved patient outcomes (e.g., improved school attendance) and to decreased healthcare utilization.

## Abbreviations

ADD: Attention Deficit Disorder; ADHD: Attention-Deficit/Hyperactivity Disorder; CI: confidence interval; DSM-IV: Diagnostic and Statistical Manual of Mental Disorders, Fourth Edition; ER: Emergency room; HCP: Healthcare provider; NHIS: National Health Interview Survey; OR: Odds ratio; SDQ: Strength and Difficulties Questionnaire; U.S.: United States.

## Competing interests

PC, DM, SW, KS, and JJ are employees and shareholders of Eli Lilly and Company.

## Authors’ contributions

PC was the principle scientist for this study. PC, DM, and JJ collaboratively wrote the first draft of the manuscript. All authors reviewed and edited subsequent drafts, and read and approved the final manuscript.
